# Fate of Fe_3_O_4_@NH_2_ in soil and their fixation effect to reduce lead translocation in two rice cultivars

**DOI:** 10.1002/fsn3.1651

**Published:** 2020-06-09

**Authors:** Chenlu Chu, Chenhao Lu, Jian Yuan, Changrui Xing

**Affiliations:** ^1^ Key Laboratory of Grains and Oils Quality Control and Processing College of Food Science and Engineering Collaborative Innovation Center for Modern Grain Circulation and Safety Nanjing University of Finance and Economics Nanjing China

**Keywords:** Fe_3_O_4_@NH_2_, fixation, lead, *Oryza sativa L*, soil

## Abstract

The fate of nanoparticles in the ecological chain of agriculture has been concerned as their potential pollution and biological effect to humans with rapid development and massive emission of nanomaterials. Here, we found that two rice cultivars (*Oryza sativa L*) have different heavy metal accumulation results in the roots and shoots after 15 days growth. Two rice cultivars (*Oryza sativa L*), grown in soil containing magnetite (Fe_3_O_4_@NH_2_) nanoparticles and heavy metal simultaneous, showed less Pb uptake in the roots and shoots, compared with that without Fe_3_O_4_@NH_2_ added. The shape and magnetic properties of Fe_3_O_4_@NH_2_ have no obvious change; however, the transmission electron microscope (TEM) results showed the shell of Fe_3_O_4_@NH_2_ could be broken in the process of interaction with soil. These results suggested that magnetite nanoparticles, such as Fe_3_O_4_@NH_2_, could potentially be used as the recyclable heavy metal fixation materials for alleviating heavy metal poisoning to plant.

## Graphical abstract

More Pb, Cd, and As accumulation in rice (*Oryza sativa* L) roots than shoots. The Si coating shell protected the nanoparticles shape from degradation in acid condition and soil environment. Pb taken up by rice (*Oryza sativa* L) from soil could be reduced based on the fixation effect of Fe3O4@NH2.

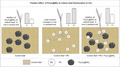

## 1 INTRODUCTION

The application of nanotechnology recently has increased in electronics, optics, catalysts, coatings, paints, pigments, medical, and energy (Stark, Stoessel, Wohlleben, & Hafner, 2015). According to incomplete statistics, the annual global production of Fe and Fe oxides has reached 42,000 tons/year in 2010. Most of them are likely to be disposed in landfills, soils, and sewage sludge (Keller, McFerran, Lazareva, & Suh, 2013). However, 60% of sewage sludge, such as biosolids, is reused to the land in the United States and United Kingdom (Unrine et al., 2010). Therefore, land is expected to be an ultimate sink for most of nanoparticles (NPs). Thus, these problems raise the concerns about potential pollution and effects of these NPs in agricultural soil and human exposure from consumption. Significant release of huge engineered nanomaterials to terrestrial ecosystem press the need to evaluate their fate and effects in soil.

Previously studies have shown that CuO NPs and Fe_3_O_4_ NPs could be absorbed from the roots and translocated to shoots in plant *Elsholtzia splendens* and rice under hydroponic conditions(Peng et al., 2015; Shi et al., 2014; Zhu, Han, Xiao, & Jin, 2008). However, due to the uncertainty and complexity of soil system, different NPs have variously fate and transformation behavior in soil. CuO NPs could be uptake directly from wheat shoots. ZnO NPs dissolved by the roots exudates to Zn^2+^ and translocated (Dimkpa et al., 2013). Also, under field conditions, ZnO NPs dissolved, inducing the uptake of Zn by wheat. The TiO_2_ NPs were also found in the vacuole of cortex cells (Du et al., 2011). This root‐to‐fruit translocation was also found in cucumber for TiO_2_ NPs (Servin et al., 2013). Likewise, the CeO_2_ NPs could be uptake and translocated from soil to roots in different plants, including corn and soybean (Hernandez‐Viezcas et al., 2013; Priester et al., 2012; Zhao et al., 2012).

However, some conflicting results were also reported. Zhu et al. have found Fe_3_O_4_ NPs and Al NPs shown no absorb in soil‐plant system and translocation in the plants grown due to their attachment with sand or soil grains. Zahra et al., (2015) have found Fe_3_O_4_ NPs in *Lactuca sativa* shoots confirmed by *SEM* image & EDX spectra. These results indicated that the environmental behavior of NPs was depending on materials (size, crystal phase, and surface coating), plant species, and soil type.

Numerous researches have been reported for Fe_3_O_4_ or its functional materials as the carrier uptake by cells, animals, and human for in vivo imaging, targeted therapy and heavy metal removal effect, which have been widely researched in the liquid phase or culture medium system (Li et al., 2018; Smith & Gambhir, 2017; Sun et al., 2018). Many metal oxide nanoparticles (NPS) are nontoxic and low‐cost materials which have also been researched as heavy metal adsorbents widely. Except ease of recovery and reusability, they have minimized environmental impact and unique advantages including large surface area and high surface activities due to the size‐quantization effect (Lee, Zaini, & Tang, 2017). In general, the process and mechanism of metal adsorption on Fe_3_O_4_‐NPS could be explained from surface interaction, including metal complexation, electrostatic interactions, micro precipitation, and ion exchange (Pu et al., 2018). The chemical reaction or cation‐exchange reaction between Fe_3_O_4_‐NPS and the metal ions are involved. And acid–base conditions (protonation and deprotonation process), temperature, and the chemistry of the metal ion (electronegativity and ionic radius) may be responsible and are important for the sorption selectivity (Sun, 2018). However, the fate of Fe_3_O_4_‐based NPs in the soil and their absorption behavior with heavy metal under more environmentally relevant conditions are still lacking.

Crops could enrich heavy metal from polluted farmland. Rice (*Oryza sativa L*.) is a staple food crop in the world, which was easily poisoned by many toxic heavy metals from the soil. A comprehensive study of rice plants cultivated in soils with different heavy metal pollution and iron oxide NPs deposition is important for better understanding the fate of iron oxide NPs and their fixation effect to reduce heavy metal translocation in plant. In this article, we have firstly targeted three heavy metals (Pb, Cd, and As) transfer process from soil to roots in two rice cultivars. Then, Fe_3_O_4_@NH_2_ were chosen and studied its interdict effect of heavy metal adsorption for Pb in the soil environment. This research was valuable for understanding the fate of NPs and the potential value of pollution control.

## 2 METHODS

### 2.1 Soil source and characteristic

Organic farm soil was obtained from Nanjing, CA. The soil was first air‐dried at room temperature and then cleaned by removing impurity including plant roots and stone. The dried soil was grinded and sieved through mesh sizes of 20 and stored at 4°C before use. Sieved soil was characterized, and the results were shown in Table S1.

The pH of the soil was 7.27. The total content of Pb, Cd, and As in the tested soil was 21.39, 0.12, and 4.33 mg/kg. This represented no Pb, Cd, and As contamination, which have not exceeded the allowable limit of 80, 0.3, and 20 mg/kg stipulated by the China Risk Control Standard for Soil Contamination of Agricultural land (GB 15618–2018). So this soil was treated as a control soil and suitable for simulation of heavy metal pollution and remediation.

### 2.2 NPs synthesized and characterization

The synthesized process of Fe_3_O_4_ NPs was based on a solvothermal method reported by Liu with some modifications (Liu et al., 2009). Fe_3_O_4_@NH_2_ were synthesized in our group by the method as described before (Sun et al., 2018). The size, and morphology were characterized by TEM, *SEM*, XRD, XPS, and FTIR.

### 2.3 Spiking of soil with Fe_3_O_4_@NH_2_ and rice cultivation in simulated polluted environment

2.5 g Fe_3_O_4_@NH_2_ were mixed as a powder into 20 kg soil mechanically 20 min with a mixer and transferred to four flowerpots with 40 cm depth. To simulate exogenous pollution of Pb, 25 ml Pb containing solution was added into the soil to obtain soil with 0, 0.1, 0.2, and 0.4 mg/kg Pb polluted. More water was added to make sure the possible homogeneity of heavy metal distribution and moist condition for rice growth. No base fertilizer was applied and the treated soil was set for 1 day before transplantation and could be regarded as unaged soil. After germination, the seedlings were transplanted to soil in different pots and grown in natural environment for rice growth and development. The moisture condition was monitored every day, and the water was supplemented to maintain the soil moisture. Two popular cultivar of Japonica Rice (*Oryza sativa L*.), Nanjing 46 and Nanjing 9108, were simultaneously planted in each flowerpot in order to evaluate the difference of rice cultivar. Each pot and rice was labeled. In the same way, Nanjing 46 and Nanjing 9108 were also planted in another four pots without Fe_3_O_4_@NH_2_ added as the control group. In order to investigate Cd and As accumulation in the roots and shoots, Cd and As were also added into the soil in different pots according to the previously described method. Each experiment was repeated three times.

After 15 days growth from seed, the absorption and transportation of Pb in the roots and shoots of rice were collected and detected by ICP‐MS.

The rice samples were first collected by cutting the shoots from the soil surface, and the obtained roots were carefully washed to remove the soil with water. The weights of shoots and roots were recorded. The concentration of rice roots and shoots were detected by ICP‐MS method, which has been described in our laboratory before (Sun, 2018). The sample was digested in the following steps. 0.2 ~ 0.5 g samples were weighed and then digested in tube with 3 ml 65% HNO_3_ and 1 ml 30% H_2_O_2_ according to the standard process. The obtained solution was heated to remove acid solution, and the volume was adjusted to 10 ml. The obtained solution was filtrated through 0.22 μm and tested by ICP‐MS. At the same time, blank experiment was also prepared.

### 2.4 Material recycling and characterization

Fe_3_O_4_@NH_2_ in the soil were recycled by magnetic separation and the obtained material containing some soil impurity, for example intrinsical Fe oxides. The recycled materials were characterized by TEM, *SEM*, XRD, XPS, and FTIR.

## 3 RESULTS

### 3.1 Synthesis and characterization of NPs

**Figure 1:**
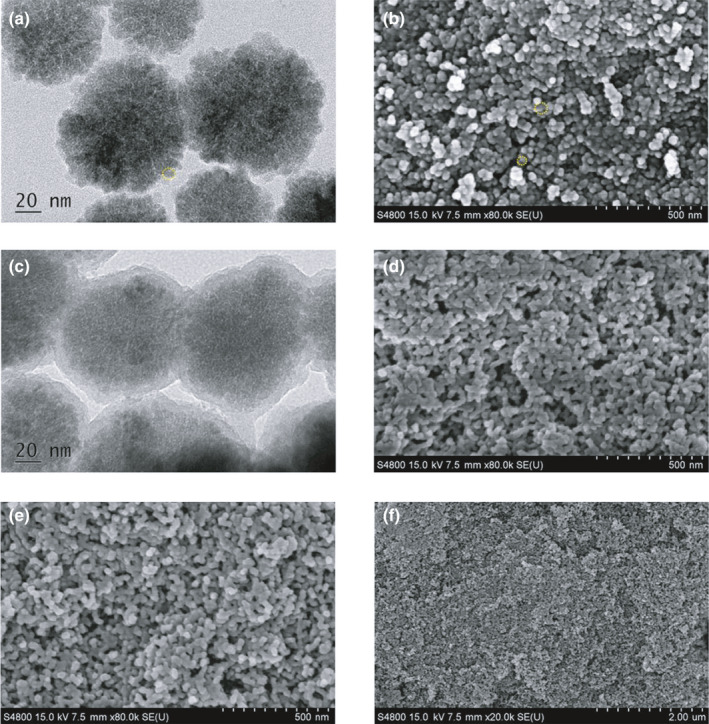
The TEM and *SEM* analysis of Fe_3_O_4_ NPs (a) and (b), Fe_3_O_4_@NH_2_ NPs (c) and (d), and Fe_3_O_4_@NH_2_ NPs with 0.01 M HCl treatment for 15 days (e) and (f)

Fe_3_O_4_ NPs, with diameter of about 100 nm, indicated that the magnetite NPs consisted of nanocrystals with 5–10 nm connected with each other loosely by amorphous matrix (Figure 1a,b). The zeta potential ξ of the magnetite particles was −21.4 mV (tested at 10 μg/ml concentration), and this negative charge density was caused by the Na_3_Cit. The citrate groups could coordinate with Fe^3+^ strongly and offer good hydrophilicity. The morphology of prepared Fe_3_O_4_@NH_2_ NPs, synthesized by sol–gel process, was examined by TEM techniques (Figure 1c). We could see that the silica shells were successfully coated on Fe_3_O_4_ with 20 nm thickness. Fe_3_O_4_@NH_2_ were very stable and intact after 0.01 M HCl treatment for 15 days (Figure 1e,f), compared with that before acid treated (Figure 1d).

XPS was also employed to analyze the chemical compositions of Fe_3_O_4_ NPs and Fe_3_O_4_@NH_2_ NPs (Figure S1). The peaks at 103.8 eV, 154.1 eV, and 532.08 eV are assigned to Si2p, Si2s, and O1s, related to the silica and oxide. The peak of N1s of Fe_3_O_4_@NH_2_ NPs indicated that –NH_2_ groups are successfully coated on the surface of Fe_3_O_4_ NPs. At the same time, Fe element (Fe2p) was disappeared in this coating process. In the XPS of Fe_3_O_4_ NPs, two major components with typical peaks at 710.7 and 724.3 eV were assigned to Fe2p_3/2 and_ Fe2p_1/2_ (Figure S1B). The main component of O1s transition at 532.1 eV was attributed to Si–O bonds, and the peak of Fe_3_O_4_ at lower binding energy, near 530.1 eV, could be a contribution of Fe–O (Figure S1C). These results are in accordance with values reported for metallosiloxane (Me–O–Si) covalent bonds (Amouzou, Fourdrinier, Maseri, & Sporken, 2014).

XRD of the Fe_3_O_4_ (Figure S2) contained the six characteristic peaks at 2θ of 30.14°, 35.6°, 43.26°, 57.1°, and 62.7°, which are marked by their corresponding indices (220), (311), (400), (511), and (440), respectively (Shi et al., 2018). Compared with Fe_3_O_4_, Fe_3_O_4_@NH_2_ showed similar XRD pattern except that an extra broad band appeared at 2θ of 23.1°. The extra broad band was attributable to the amorphous SiO_2_ coating on the surface of Fe_3_O_4_, which was consisted with the previous results (Deng, Qi, Deng, Zhang, & Zhao, 2008; Zhang et al., 2013). The consistency of the crystal structure of Fe_3_O_4_ NPs and Fe_3_O_4_@NH_2_ NPs indicated no change in the modification process.

In the FTIR spectra of Fe_3_O_4_ NPs (Figure S3), the peak around 583 cm^−1^ was caused by the typical Fe–O vibration. The peak of 1,084 cm^−1^ corresponded to the Si–O–Si asymmetric stretching vibration, proving the formation of silica shells on the surface of Fe_3_O_4_. These results were consistent with the conclusions of TEM, XRD, and XPS.

### 3.2 Heavy metal accumulation in roots and shoots

**Figure 2:**
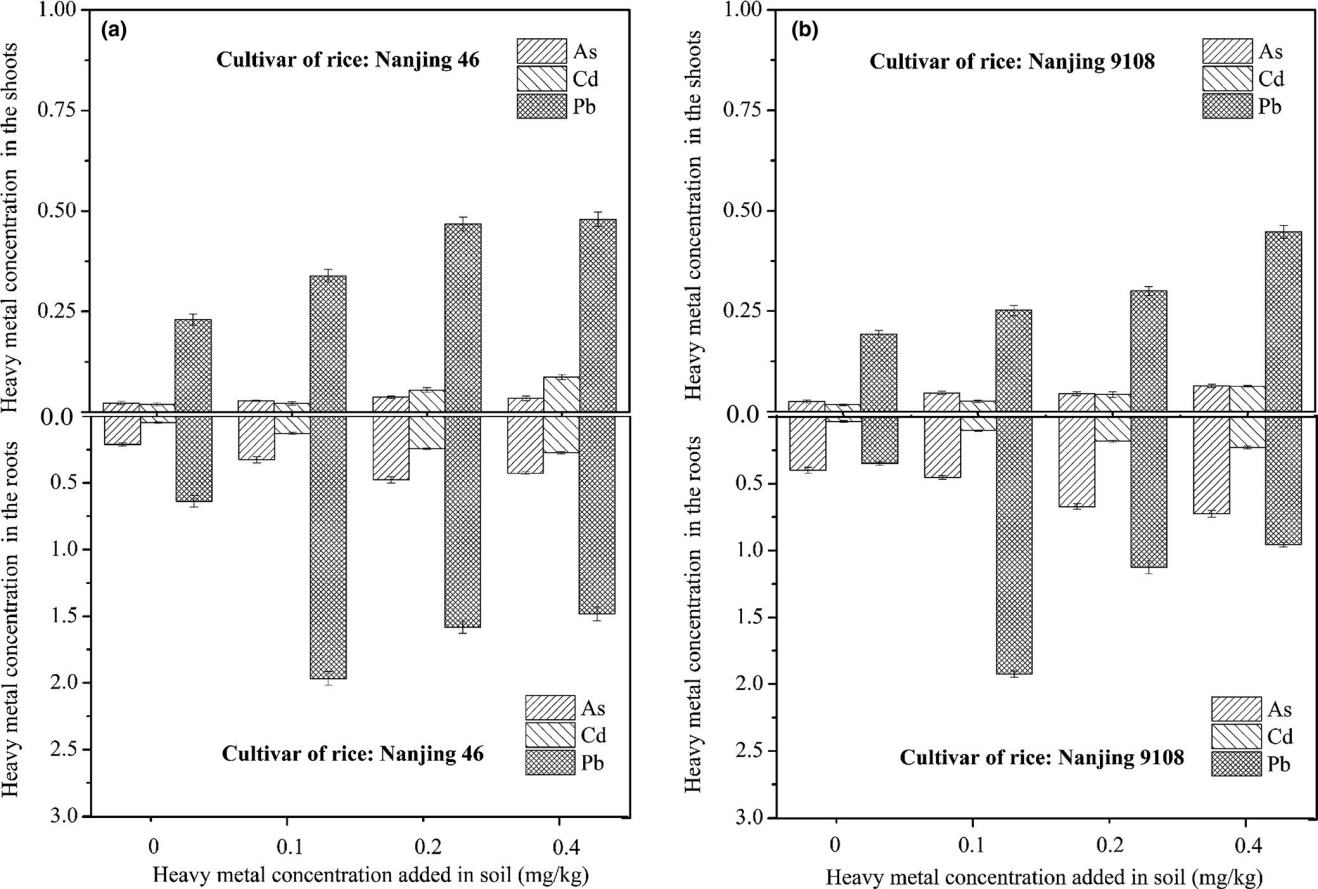
The concentrations of Pb, Cd, and As in the roots and shoots of Nanjing 46 (a) and Nanjing 9108 (b) obtained from heavy metal polluted soil

The accumulation effects (Figure 2) of Pb, Cd, and As in the roots and shoots of rice *Oryza sativa L*. were obtained and analyzed. For cultivar of rice Nanjing 46, higher Pb concentrations of 1.97, 1.58, and 1.48 mg/kg were found in rice roots and 0.34, 0.47, and 0.48 mg/kg in shoots, obtained from sample treated with 0.1, 0.2, and 0.4 mg/kg Pb. In the case of Nanjing 9108, the concentrations of Pb in the roots were 1.92, 1.13, and 0.96 mg/kg and 0.25, 0.3, and 0.45 mg/kg in shoots. Pb, Cd, and As concentrations in the roots and shoots of two rice cultivars have different degrees of increase with heavy metals added in the soil. Pb in the roots and shoots with higher concentration than Cd and As, which may be caused by high background value. In the roots, the Pb concentrations were not increased consistently with Pb contents added in the soil. The reasons for these different results were complex and might due to the soil type used, genotypic differences, the growth stage, added Pb amount, and etc (Ashraf et al., 2020; Liu, Ma, Wang, & Sun, 2013; Liu, Zhang, Zhao, Sun, & Liu, 2017).

As for samples of Nanjing 46 collected from soil treated with 0.1, 0.2, and 0.4 mg/kg As (Figure 2a), a relative concentration increase of 54%, 125%, 101% in the roots, and 27%, 68%, 55% in the shoots were observed, compared with the control. In Nanjing 9108, the values of 0.45, 0.67, and 0.73 mg/kg of As were observed in the roots of treated group, which were 1.13 ~ 1.8 times higher than the control (0.399 mg/kg). As to the As concentration in shoots in the same condition, the values were 0.05, 0.05, and 0.06 mg/kg, which were 1.88–2.56 times higher than the control (0.025 mg/kg).

From the above results, we can see that the concentrations of Pb and As enriched in the roots were higher than that enriched in the shoots. Differential trends were observed in the case of Pb and As accumulation by the two cultivar rice, such as Nanjing 46 have relative higher Pb absorption ability than Nanjing 9108, yet inverse for As.

The concentrations of Cd of Nanjing 46 were 0.13, 0.243, and 0.274 mg/kg in the roots of treated samples and 0.021, 0.055, and 0.087 mg/kg in the shoots of three treated samples. As for the Nanjing 9108, the corresponding values were 0.102, 0.183, and 0.227 mg/kg in the roots and 0.026, 0.043, and 0.063 mg/kg in the shoots (Figure 2b). For Pb translocation and accumulation in the roots of two rice cultivars, the detected concentrations were most among three spiked heavy metals, which may be relevant with the concentrations and available state of heavy metals in tested soil. The original Pb concentration in soil is far higher than that of Cd and As. The concentrations were similar for Cd and As in the shoots of Nanjing 9108, which were far lower than that of Pb.

Significant interaction existed between rice genotype and soil condition leading different trends of heavy metal concentrations of rice. Zeng, Mao, Cheng, Wu, & Zhang, (2008) have been researched genotypic and environmental variation in Cr, Cd, and Pb concentration in rice, based on 138 genotypes grown in three contaminated soils. For 138 rice genotypes, a remarkable result was that the mean values of Pb in grain and straw are higher than that of Cd in the three types of soil (slightly contaminated soil, moderately contaminated soil, and highly contaminated soil). The genotypic effects play more important role on the variation of Cr, Cd and Pb concentrations than the soil effect. The findings in our experiment were consistent with these reports.

Rieuwerts et al., 2006 have reported that characteristics of soil would influence on the extractability of Cd, Pb, and Zn in soils. The estimation of CaCl_2_‐extractable Cd, Pb, and Zn was mainly be affected by pH. In that article, the pristine Pb concentration is from 11.6 to 176.3 mg/kg with mean value of 60.3 mg/kg. The pristine Cd concentration in soils was from 0.10 to 1.38 mg/kg with mean value of 0.82 mg/kg. However, the percentage of total metal extracted by 0.01 M CaCl_2_ was from 0.28 to 82.98 mg/kg for Cd and from 0.003 to 7.00 mg/kg for Pb. This indicated Pb in the soil was uneasy to be extracted and absorbed by the plant due to a long‐term fixation (Rieuwerts, Ashmore, Farago, & Thornton, 2006). However, some exogenous heavy metal pollution in soil existed as free state, which could be easily transferred to the plant, and only 28% Cd has been fixated after 850 days (Buekers et al., 2007). In our results, all three heavy metals showed increased concentrations and they are easily be enriched by rice in the soil used in the experiment. Pb in the roots and shoots with higher concentration than Cd, which may be due to no aging process and high background value. In the next experiments, we aim to using Fe_3_O_4_@NH_2_ to fix exogenous Pb discharged into the soil and explore their effect to the Pb transport in early stage of rice growth.

### 3.3 Fate and fixation effect of Fe_3_O_4_@NH_2_ in soil

Fe_3_O_4_ based nanoparticles have been widely used for absorption heavy metal in liquid solution. However, little was focus on their absorption ability in soil. Due to the complexity of soil, the morphology of most nanoparticles, especially uncovered metal nanoparticles, has changed in the process of interaction with matrix of soil and individual nanoparticles are difficult to separate from the bulk soil (Theng & Yuan, 2008).

First, the ratio of Fe3O4@NH2 to soil was （0.0125%）selected based on references and the maximal adsorption capacity analysis. In Rico's review, we can see that in most of experiments different nanoparticles concentrations were used. It was very hard to regulate an uniform ratio as different property of nanoparticles (Rico, Majumdar, Duarte‐Gardea, Peralta‐Videa, & Gardea‐Torresdey, 2011). As for the application of iron oxide nanoparticles, in Zhu's report, they have used 0.5 g/L Fe_3_O_4_ in an aqueous medium (the ratio was 0.05%) to investigate the uptake of manufactured iron oxide nanoparticles by pumpkin (Zhu et al., 2008). In the soil, we could anticipate that the low mobility, interferences from soil, and competitive metal ions will change the adsorption capacities. Consideration of pre‐existed heavy metals in soil and poor mobility of added Fe_3_O_4_@NH_2_, this ratio was acceptable.

**Figure 3:**
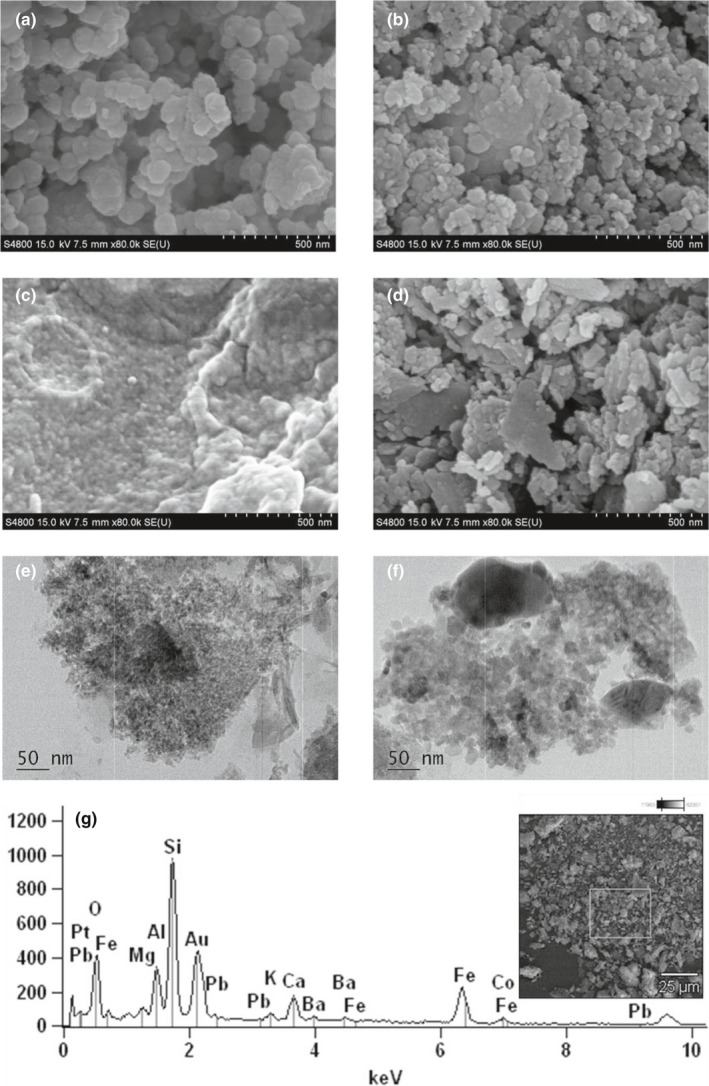
*SEM* of Fe_3_O_4_@NH_2_ NPs treated with 0.01 M PBS (a) and soil leaching fluid (b) for 15 days. *SEM* of Fe oxides separated from soil (c). *SEM* (d), TEM (e) and (f) and *SEM*‐EDX (g) of Fe_3_O_4_@NH_2_ NPs recycled from soil

Fe_3_O_4_@NH_2_ were stable as the coating shell of SiO_2_ in the surface. In previous results, we have found only 5.2% Fe element could be found in 1M HCl after 24 hr (the concentration of Fe_3_O_4_@NH_2_ was 10 mg/ml). Here, we have treated Fe_3_O_4_@NH_2_ with 0.01 M PBS and soil leaching fluid for 15 days. *SEM* results (Figure 3a,b) showed that the nanoparticles were stable and the Si shell coating maintained the shape of the structure of Fe_3_O_4_. In soil, Fe oxides also existed and could be separated by magnet (Figure 3c). *SEM* results showed they have the similar surface morphology. The results showed Fe_3_O_4_@NH_2_ were still complete and could be recycled after short‐period coexistence with soil matrix (Figure 3d). However, the fate and the potential co‐transport risks in long time need more in‐depth studies as some reports have shown nanoparticles could be transported from soil to plant. Interactions among soil, root exudates, microbial community, and nanoparticles for remediation will be very complicated and high‐risk potentially (Chen et al., 2019; Ma, White, Zhao, Zhao, & Xing, 2018).

To investigate the fate of Fe_3_O_4_@NH_2_ in soil with heavy metal polluted, Fe_3_O_4_@NH_2_ have been mixed into the soil and rice was planted for 15 days after Pb polluted. Pb polluted soil without Fe_3_O_4_@NH_2_ was prepared as the control sample.

The high magnification *SEM* of Fe oxides and Fe_3_O_4_@NH_2_ mixture (Figure 3d), recycled from soil by a strong magnet, showed that most of the Fe_3_O_4_@NH_2_ maintained their morphology. As interference from complex soil, XPS results of Pb element (Pb4f) from Fe_3_O_4_‐NH_2_‐Pb (Figure S1D) could not be observed obviously between 135 eV to 150 eV as reported by others (Bai, Feng, Hua, Zhou, & Shi, 2015; Rijith, Anirudhan, Sumi, & Shripathi, 2015). This undetected peak may due to the low Pb concentration and interference from high abundance of Si. This masking effect were also observed in XRD results (Figure S2), this sharp peak of Fe_3_O_4_@NH_2_ recovered from soil was complete different with that of original Fe_3_O_4_@NH_2_, which was caused by the complexes of elements in soil (Singh & Agrawal, 2012). The FTIR result (Figure S3) showed very similar peaks with that of Fe_3_O_4_, indicating the existence of Fe_3_O_4_@NH_2_ recycled from soil after interacted with materials in soil.

On the other side, TEM of Fe_3_O_4_@NH_2_ recovered from soil is shown (Figure 3e,f). The integrality of NPs has been partially destroyed from the interaction with soil. The silica coating has been broken and small nanocrystals with 5–10 nm have been random aggregated together. This break could not observed from *SEM*. Some very big particles (Figure 3f) may be the Fe oxides recovered from soil. The *SEM*‐EDX analysis results have been also shown (Figure 3g). Elements in weight percent were O (34.18%), Si (25.41%), Fe (18.02%), Al (8.32%), Ca (5.00%), and Pb (2.18%). As the element complex in soil, lead EDX peaks are contributed by Pb containing Fe oxides from soil and Fe_3_O_4_@NH_2_ with adsorption of Pb.

As Fe oxides existed in the soil could be also separated by magnet with Fe_3_O_4_@NH_2_ simultaneously, direct quantification of Pb on Fe_3_O_4_@NH_2_ was difficult. To evaluate the Pb fixation ability of Fe_3_O_4_@NH_2_ in soil, the obtained roots and shoots were tested to found the Pb fixation effect in soil with and without Pb pollution.

**Figure 4:**
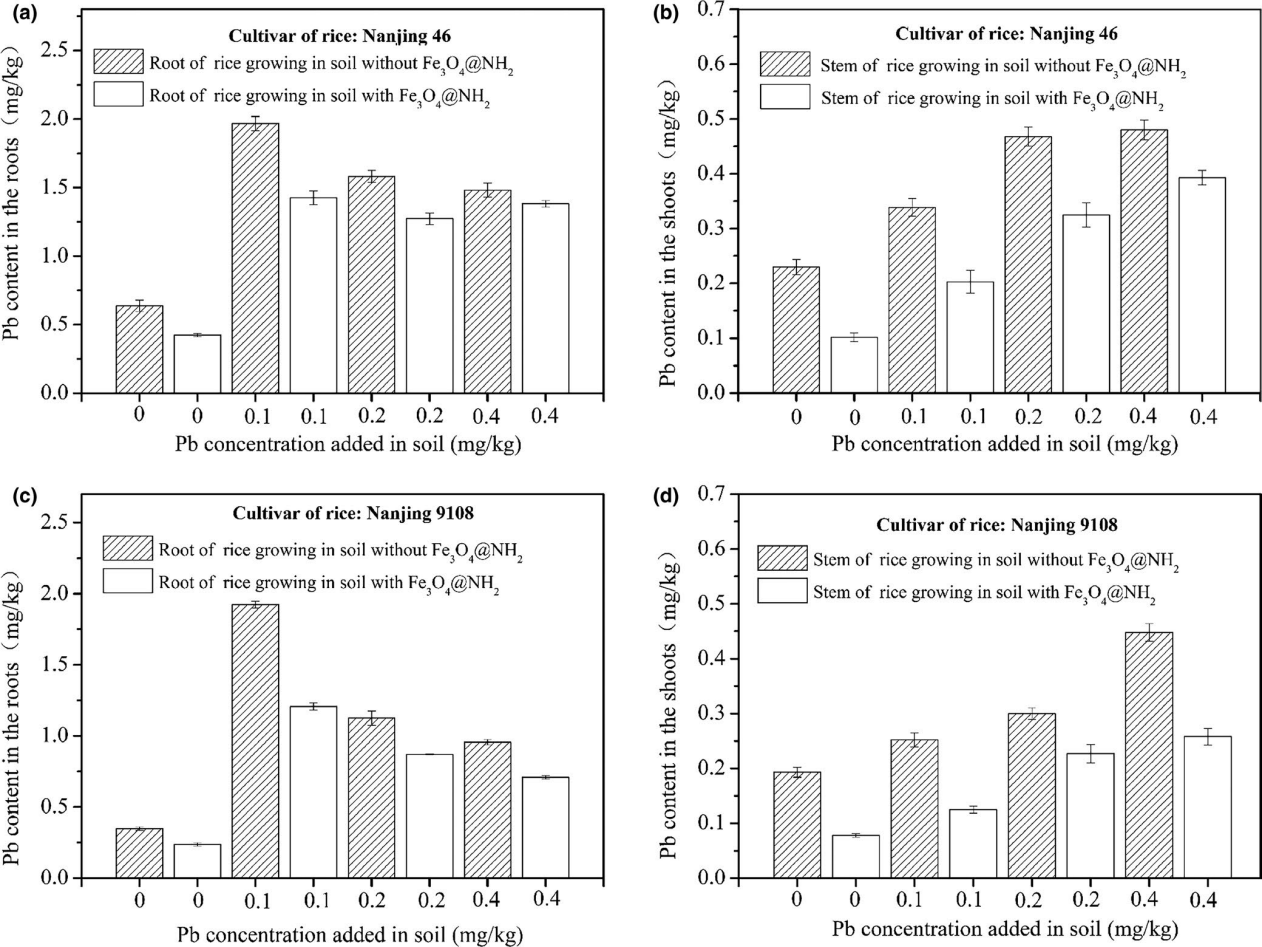
The Pb concentration in the roots and shoots of Nanjing 46 (a) and (b) and Nanjing 9108 (c) and (d) obtained from soil containing magnetite (Fe_3_O_4_@NH_2_) nanoparticles with or without Pb added

We could notice that, for Nanjing 46 and 9108, the Pb concentration in the roots and shoots are all both higher than that of control set (Figure 4). And the concentration of Pb in the roots are two to fourfold than that in shoots. With the increasing Pb added in the soil, the uptaken Pb was decreased in the roots and increased in the shoots. As to the fixation effect of Fe_3_O_4_@NH_2_, these results showed that Pb concentrations in the roots and shoots obtained from the soil with Fe_3_O_4_@NH_2_ added are lower than that without Fe_3_O_4_@NH_2_. The experiments showed that Fe_3_O_4_@NH_2_ have immobilized some Pb in the soil and reduced the amount of Pb in the rice. Considering the soil itself could absorption of Pb added and pristine Pb in soil could also be absorbed by plant, it was very hard to calculate the exact proportion of exogenous Pb, which have been enriched by the two rice. Furthermore, as the designed ratio of Fe_3_O_4_@NH_2_ to soil (2.5 g/20 kg) was small, the fixation effect of Fe_3_O_4_@NH_2_ for Pb in soil was effective.

In summary, we demonstrated that Fe_3_O_4_@NH_2_ particles, with percent of 0.0125%, added into the soil can immobilize heavy metals in soil environment and reduce the transfer in two rice cultivars (*Oryza sativa L*). Unlike in an aqueous medium, this particles have little mobility. Interactions between Fe_3_O_4_@NH_2_ particles and soil could lead the broken of the Si coating shell, although most of the particles still maintained showed by *SEM*. The fixation of Pb by the particles is obviously, reflected by the decreased Pb concentration in the roots and shoots of rice. This magnetic particles could be recycled and have huge potential in heavy metal fixation. Such studies are useful to understand the different impacts of nanomaterials on ecological chain of agriculture and public health.

## CONFLICT OF INTERESTS

Authors declare no conflict of interest.

## Supporting information

Appendix S1
