# Supplementary material for: Fate of Fe3O4@NH2 in soil and their fixation effect to reduce lead translocation in two rice cultivars
Source: Food Sci Nutr. 2020 Jun 9;8(7):3673–81. doi: 10.1002/fsn3.1651 (PMC7382116; doi:10.1002/fsn3.1651)
Supplement: Supplementary file 1 — Appendix S1 [file FSN3-8-3673-s001.docx]

*Electronic Supplementary Information*

**Fate of Fe_3_O_4_@NH_2_ in soil and their fixation effect to reduce lead translocation in two rice cultivars**

Chenlu Chu, Chenhao Lu, Jian Yuan^*^, Changrui Xing^*^

College of Food Science and Engineering/Collaborative Innovation Center for Modern Grain Circulation and Safety/Key Laboratory of Grains and Oils Quality Control and Processing, Nanjing University of Finance and Economics, Nanjing 210023, People’s Republic of China

⁎ Corresponding authors. Tel: +86 025 86718509

yjian_nj@163.com

[da_rui12345@163.com](mailto:da_rui12345@163.com)

**Table S1 Properties of soil used in the experiment (mg/kg)**

| soil texture | pH | available phosphorus | soil potassium | total nitrogen | organic matter | Pb | Cd | As |
| --- | --- | --- | --- | --- | --- | --- | --- | --- |
| sandy soils | 7.27 | 0.79 | 16.63 | 880.0 | 12800.0 | 21.39 | 0.12 | 4.33 |


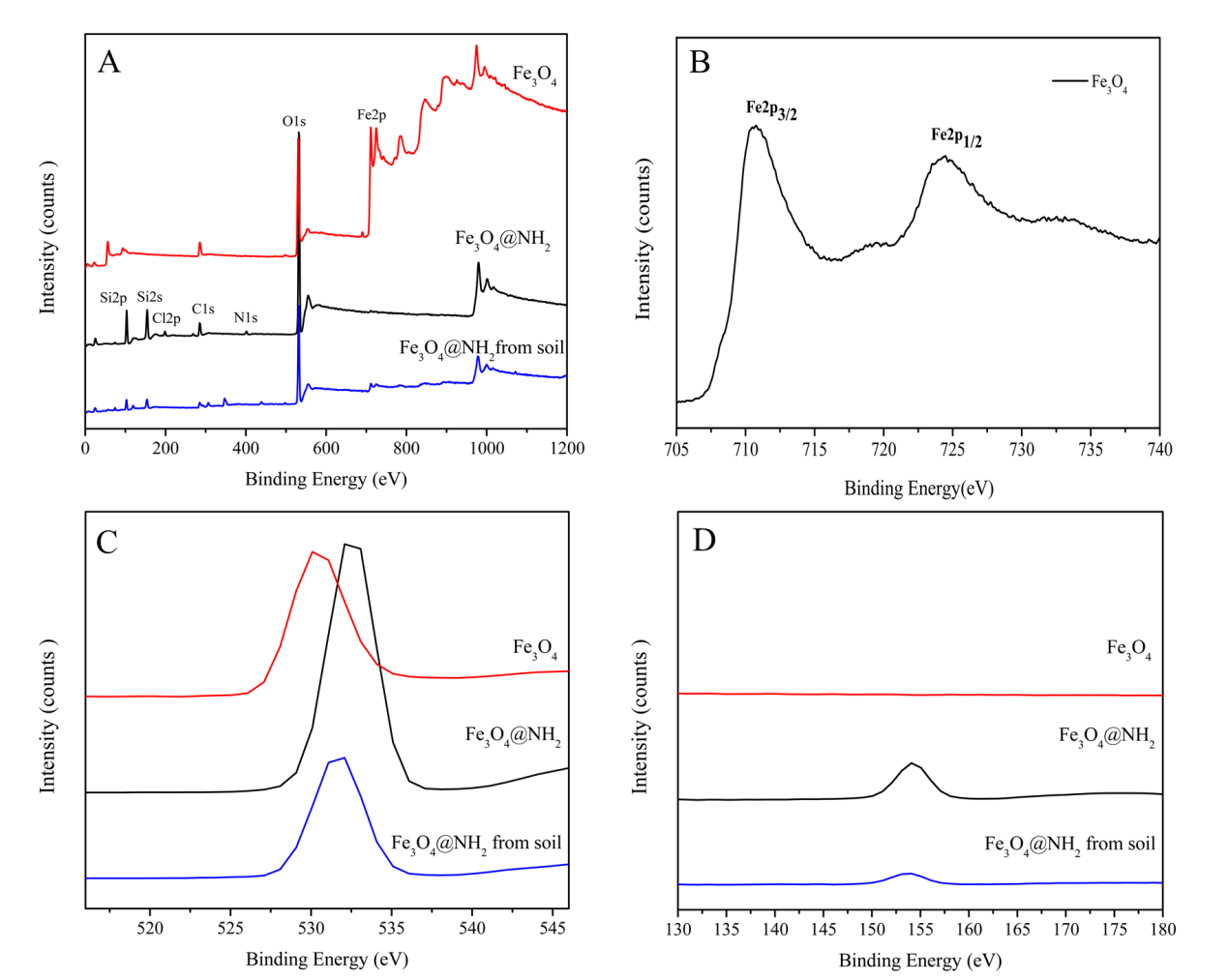


**Fig.S1** XPS profile of Fe_3_O_4_, Fe_3_O_4_@NH_2_, and Fe_3_O_4_@NH_2_ recycled from soil (A), and the high-resolution spectra of Fe2p (B), O1s (C) and Pb4f (D)


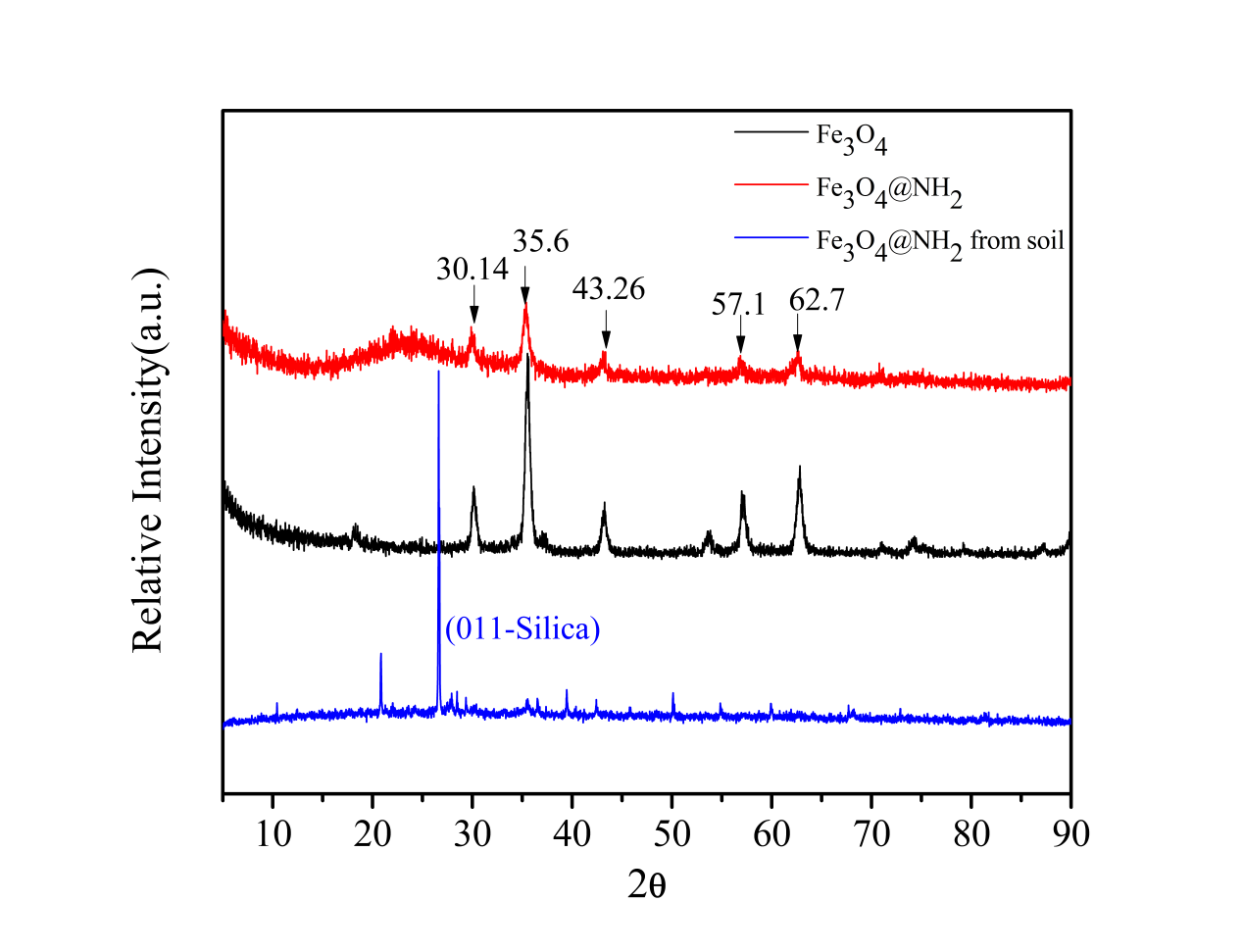


**Fig.S2** XRD profile of Fe_3_O_4_, Fe_3_O_4_@NH_2_, and Fe_3_O_4_@NH_2_ recycled from soil


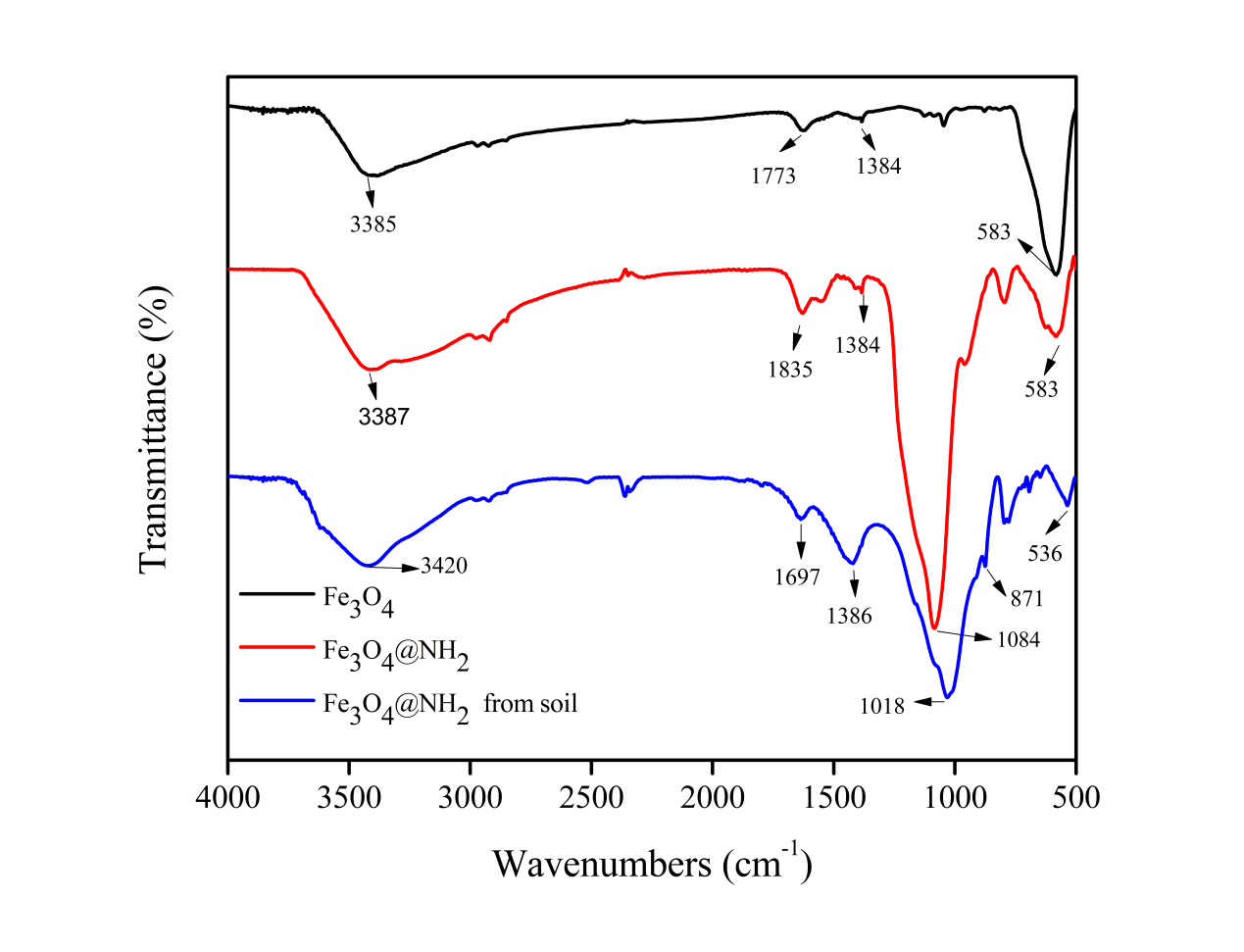


**Fig.S3** FTIR profile of Fe_3_O_4_, Fe_3_O_4_@NH_2_, and Fe_3_O_4_@NH_2_ recycled from soil
